# Predicting admissions and time spent in hospital over a decade in a population-based record linkage study: the EPIC-Norfolk cohort

**DOI:** 10.1136/bmjopen-2015-009461

**Published:** 2016-01-20

**Authors:** Robert Luben, Shabina Hayat, Nicolas Wareham, K T Khaw

**Affiliations:** 1 Department of Public Health and Primary Care, University of Cambridge, Cambridge, UK; 2 Medical Research Council Epidemiology Unit, University of Cambridge, Cambridge, UK

**Keywords:** EPIDEMIOLOGY, HEALTH SERVICES ADMINISTRATION & MANAGEMENT, PUBLIC HEALTH

## Abstract

**Objective:**

To quantify hospital use in a general population over 10 years follow-up and to examine related factors in a general population-based cohort.

**Design:**

A prospective population-based study of men and women.

**Setting:**

Norfolk, UK.

**Participants:**

11 228 men and 13 786 women aged 40–79 years in 1993–1997 followed between 1999 and 2009.

**Main outcomes measures:**

Number of hospital admissions and total bed days for individuals over a 10-year follow-up period identified using record linkage; five categories for admissions (from zero to highest ≥7) and hospital bed days (from zero to highest ≥20 nights).

**Results:**

Over a period of 10 years, 18 179 (72.7%) study participants had at least one admission to hospital, 13.8% with 7 or more admissions and 19.9% with 20 or more nights in hospital. In logistic regression models with outcome ≥7 admissions, low education level OR 1.14 (1.05 to 1.24), age OR per 10-year increase 1.75 (1.67 to 1.82), male sex OR 1.32 (1.22 to 1.42), manual social class 1.22 (1.13 to 1.32), current cigarette smoker OR 1.53 (1.37 to 1.71) and body mass index >30 kg/m² OR 1.41 (1.28 to 1.56) all independently predicted the outcome with p<0.0001. Results were similar for those with ≥20 hospital bed days. A risk score constructed using male sex, manual social class, no educational qualifications; current smoker and body mass index >30 kg/m², estimated percentages of the cohort in the categories of admission numbers and hospital bed days in stratified age bands with twofold to threefold differences in future hospital use between those with high-risk and low-risk scores.

**Conclusions:**

The future probability of cumulative hospital admissions and bed days appears independently related to a range of simple demographic and behavioural indicators. The strongest of these is increasing age with high body mass index and smoking having similar magnitudes for predicting risk of future hospital usage.

**Strengths and limitations of this study**

- Prospective cohort design, with a population of community-dwelling participants enabling us to examine hospital activity with clearly defined population denominators.
- Large study size of middle aged and older men and women with a long follow-up time and detailed measurements of demographic and behavioural indicators.
- It was not possible for us to infer causal links between the lifestyle factors and hospital admissions.
- We were not able to examine non-National Health Service hospitals and clinics where study participants paid for treatment.

## Introduction

In the UK, the number of men and women over 65 years of age was 10.8 million in 2012 and is projected to increase to 17.8 million by 2037 with those over-85s doubling in number to 3.6 million.^1^ Two-thirds of people admitted to hospital are over 65 years old with those over 85 years accounting for 25% of bed days.^2^ Though increasing age is associated with increased health service usage, other factors may help identify those at greatest risk of admission. Most studies examining hospital activity start from those hospitalised but are limited with respect to population denominators;^3–7^ even those that use general practice record linkage studies only include people who attended general practices while population-based studies that have measured factors prospectively prior to admission are limited.^8–13^

In this study we examined the relationship between simple and easily measurable demographic and behavioural factors to predict in a general population cohort resident in Norfolk, the future risk of use of National Health Service (NHS) hospitals over a 10-year period from 1999 to 2009, a period of relative stability for the NHS under Primary Care Trusts.

## Methods

The European Prospective Investigation into Cancer, Norfolk (EPIC-Norfolk), is a cohort of men and women aged 40–79 years living in Norfolk recruited from participating general practitioner practices between 1993 and 1998.^14^ ^15^

### Study design

A total of 25 639 participants completed a lifestyle questionnaire on recruitment and attended a clinic where weight and height and other measurements were made by trained nurses using standard protocols. Body mass index (BMI) was calculated as weight in kilograms divided by height² in square metres. The lifestyle questionnaire included questions relating to current and former employment.

Occupational social class was defined according to the Registrar General’s classification. Non-manual occupations were represented by codes I (professional), II (managerial and technical), IIIa (non-manual skilled) occupations, while manual occupations were represented by codes IIIb (manual skilled), IV (partly skilled) and V (unskilled) occupations.

Educational attainment was established using the question ‘Do you have any of the following qualifications’ followed by a list of common UK qualifications. Participants were categorised according to the highest qualification attained in three groups: those with no formal qualifications; those with formal qualifications usually associated with a school age between 16 and 18 years; and those with degree level qualifications.

Smoking status was derived from two questions each of which could be answered as yes or no: ‘Have you ever smoked as much as one cigarette a day for as long as a year?’ and for those who answered yes to the first question ‘Do you smoke cigarettes now?’

### Record linkage

Between 1999 and 2009, cohort participants were linked to hospital records using their unique NHS numbers.^16^ We used databases maintained by the East Norfolk Primary Health Care trust (PCT) an approach with the advantage that all hospital activity for Norfolk residents was captured wherever they were treated in England and Wales. The majority (95%) of admissions were to the Norfolk and Norwich University Hospitals NHS Foundation Trust (formerly Norfolk and Norwich Hospital), the remainder being admissions to other hospitals in Norfolk and neighbouring counties, community and mental healthcare trusts admissions, surgery performed in general practices and a small number of emergency admission elsewhere in the country. The PCT changed their computer systems several times over the period of study and although the data were collected from the same sources, different database systems such as Health Interlock, East Norfolk Core minimum data set (ENCORE) and others were used for linkage at different times.

Participants were also followed for mortality through linkage with the Office for National Statistics. Data from hospital records were available for inpatient episodes and outpatient visits. Records of inpatient data were organised with one row corresponding to one hospital episode. Typically patients would have several episodes for each admission. Dates of the start and end of each episode and the admission and discharge dates were included.

Each episode also had associated with it one of more International Classification of Disease V.10 (ICD10) diagnosis code and one or more OPCS Classification of Interventions and Procedures V.4 (OPCS4) procedure code. Using these data it was possible to build a fairly detailed picture of a person’s hospital stay. Outpatient data were more limited in scope, restricted to dates of a clinic visit and the specialty concerned.

Time in hospital was calculated using admission and discharge dates by summing the time between admission and discharge for each person. We used the formula one plus (discharge date minus admission date) to ensure that time in hospital for those admitted and discharged on the same day (day cases) was considered. Hospital admissions were also calculated using admission, discharge, episode start and end dates. To avoid counting immediate readmissions, where one hospital stay followed on rapidly from another, contiguous admissions were merged and counted as a single admission.

Over the 10 years of follow-up, the numbers of admissions were categorised as 0, 1, 2–3, 4–6 and ≥7. Bed days were also classified into five categories, none, day case, 1–4 nights, 5–19 nights and ≥20 nights. Three main outcomes were used: hospital admissions ≥7; Bed days ≥20 nights; and no admissions; and compared respectively with those not in those categories.

### Statistical analyses

We examined the distribution of hospital admissions by baseline descriptive data. ORs for each of the main outcomes: ≥7 hospital admissions; bed days ≥20 and no hospital admissions were calculated using unmatched logistic regression with independent variables age, smoking, BMI >30, manual social class and no educational qualifications. We then created a summary risk score, defined as the sum of five baseline risk factors dichotomised as binary categories each coded one or zero. The categories, each contributing one point were male sex, manual social class, low education level (those with no qualifications), current smoker and BMI >30 kg/m². Those with scores four and five were combined into a single category as the number with score equal to five was very low.

We used logistic regression rather than survival analysis to prevent the censoring of participants who had died, since we wished to make no distinction between non-attendance of hospital due to good health and non-attendance because of death. The number of missing values were: 53 BMI, 218 smoking status, 545 social class, 18 level of education. We examined mortality rates in the cohort by risk score stratified by age over three periods of follow-up time: 1993–1998, 1999–2004; and 1999–2009 to explore the possibility of differential mortality and therefore attrition of the population in the different risk groups which might explain some of the patterns observed. In addition, to explore the possibility of the effect of participant migration during the period under examination a sensitivity analysis was conducted on the subset of the cohort whose postcode area was Norfolk (‘NR’) at both the start and end of the period. All analyses were performed using the R statistical language (R Foundation for Statistical Computing, Vienna, Austria V.3.1.2 with packages knitr, Gmisc and IRanges) and Stata statistical software V.12 (Stata Corporation, College Station, Texas, USA).

## Results

For the current analyses, we excluded the 625 men and women from the baseline cohort who died before 1999 leaving 11 228 men and 13 786 women. Over a period of 10 years, between 1999 and 2009, 8300 (72.7%) male and 9879 (72.7%) female study participants were admitted to hospital. In total 92% of these admissions were to the Norfolk and Norwich Hospital. Descriptive characteristics of the cohort are shown in table 1. Table 2 shows the distribution of characteristics by hospital admission category. The proportion of study participants with no hospital admissions decreased monotonically across categories of age band, smoking status (never, former, current), six levels of social class, level of education (high, medium, low) and four categories of BMI while the proportion shows a monotonic increase for the same variables in the highest categories of admission. Table 3 shows similar analyses and results for increasing categories of and bed day numbers.

**Table 1:** Descriptive characteristics of men and women in the EPIC-Norfolk cohort 1993–1997 and hospital admission 1999–2009

	Men (n=11 228 44%)	Women (n=13 786 55%)
Hospital activity, 1999–2009 (n (%))		
One or more admissions	8300 (74)	9879 (72)
No admissions	2928 (26)	3907 (28)
Total hospital days, 1999–2009		
Mean±SD, full cohort	17.1±43.4	15.6±48.8
Mean±SD, excluding non-attenders	23.2±49.1	21.8±56.5
Median(IQR), full cohort	4.0 (0.0–17.0)	3.0 (0.0–13.0)
Median(IQR), excluding non-attenders	9.0 (3.0–25.0)	7.0 (2.0–21.0)
Number of admissions, 1999–2009		
Mean±SD, full cohort	4.2±16.2	3.6±16.3
Mean±SD, excluding non-attenders	5.7±18.6	5.0±19.0
Median(IQR), full cohort	2.0 (0.0–5.0)	2.0 (0.0–4.0)
Median(IQR), excluding non-attenders	3.0 (2.0–6.0)	3.0 (1.0–5.0)
Body mass index, kg/m²		
Mean±SD	26.5±3.3	26.2±4.3
Age, years		
Mean±SD	59.3±9.2	58.8±9.3
Smoking status (n (%))		
Current	1356 (12)	1548 (11)
Former	6044 (54)	4379 (32)
Never	3748 (34)	7721 (57)
Social class (n (%))		
Professional (1)	854 (8)	870 (6)
Technical (2)	4229 (38)	4720 (35)
Clerical NM (3.1)	1381 (13)	2663 (20)
Clerical M (3.2)	2781 (25)	2845 (21)
Semiskilled (4)	1466 (13)	1800 (13)
Unskilled (5)	321 (3)	539 (4)
Level of education (n (%))		
Low	3348 (30)	5782 (42)
Medium	6895 (61)	6396 (46)
High	976 (9)	1599 (12)
Body mass index (n (%))		
<24 kg/m²	2369 (21)	4616 (34)
24–27 kg/m²	4392 (39)	4216 (31)
27–30 kg/m²	2957 (26)	2608 (19)
>30 kg/m²	1486 (13)	2317 (17)

**Table 2:** Distribution of characteristics of 25 014 men and women in 1993–1997 by category of number of hospital admissions 1999–2009

	Number of hospital admissions,* 1999–2009					
	0 (n=6835 27%)	1 (n=4582 18%)	2–3 (n=6034 24%)	4–6 (n=4101 16%)	≥7 (n=3462 14%)	p Value
Total hospital days, 1999–2009						
Mean±SD	0.0±0.0	5.4±42.1	11.2±28.9	24.3±39.3	62.2±84.0	<0.0001
Median(IQR)	0.0 (0.0–0.0)	1.0 (1.0–3.0)	5.0 (3.0–11.0)	13.0 (7.0–27.0)	40.0 (22.0–73.8)	
Number of admissions, 1999–2009						
Mean±SD	0.0±0.0	1.0±0.0	2.4±0.5	4.8±0.8	16.5±41.3	<0.0001
Median(IQR)	0.0 (0.0–0.0)	1.0 (1.0–1.0)	2.0 (2.0–3.0)	5.0 (4.0–5.0)	10.0 (8.0–14.0)	
Body mass index, kg/m²						
Mean±SD	25.9±3.7	26.1±3.8	26.4±3.9	26.8±4.0	27.1±4.2	<0.0001
Sex (n (%))						
Men	2928 (26)	2012 (18)	2586 (23)	1938 (17)	1764 (16)	<0.0001
Women	3907 (28)	2570 (19)	3448 (25)	2163 (16)	1698 (12)	
Age, years						
Mean±SD	55.4±8.6	57.3±8.9	59.9±9.1	62.4±8.9	63.0±8.6	<0.0001
Age band (n (%))						
<45	481 (47)	246 (24)	186 (18)	64 (6)	55 (5)	<0.0001
45–50	1789 (41)	989 (23)	898 (21)	415 (10)	275 (6)	
50–55	1450 (35)	828 (20)	988 (24)	507 (12)	396 (9)	
55–60	1096 (28)	764 (20)	958 (25)	599 (15)	492 (13)	
60–65	877 (23)	713 (18)	980 (25)	690 (18)	636 (16)	
65–70	659 (17)	593 (15)	1018 (27)	836 (22)	732 (19)	
70–75	400 (13)	371 (12)	824 (27)	778 (25)	722 (23)	
75–80	83 (12)	78 (11)	182 (26)	212 (30)	154 (22)	
Smoking status (n (%))						
Current	751 (26)	514 (18)	665 (23)	485 (17)	489 (17)	<0.0001
Former	2558 (25)	1833 (18)	2549 (24)	1818 (17)	1665 (16)	
Never	3476 (30)	2199 (19)	2772 (24)	1754 (15)	1268 (11)	
Social class (n (%))						
Professional (1)	599 (35)	335 (19)	380 (22)	234 (14)	176 (10)	<0.0001
Technical (2)	2754 (31)	1697 (19)	2068 (23)	1348 (15)	1082 (12)	
Clerical NM (3.1)	1047 (26)	732 (18)	981 (24)	690 (17)	594 (15)	
Clerical M (3.2)	1397 (25)	1039 (18)	1375 (24)	961 (17)	854 (15)	
Semiskilled (4)	744 (23)	555 (17)	868 (27)	603 (18)	496 (15)	
Unskilled (5)	163 (19)	139 (16)	222 (26)	160 (19)	176 (20)	
Level of education (n (%))						
Low	1910 (21)	1545 (17)	2321 (25)	1815 (20)	1539 (17)	<0.0001
Medium	4122 (31)	2563 (19)	3059 (23)	1918 (14)	1629 (12)	
High	800 (31)	471 (18)	652 (25)	362 (14)	290 (11)	
Body mass index (n (%))						
<24 kg/m²	2225 (32)	1365 (20)	1660 (24)	969 (14)	766 (11)	<0.0001
24–27 kg/m²	2410 (28)	1599 (19)	2105 (24)	1349 (16)	1145 (13)	
27–30 kg/m²	1320 (24)	1008 (18)	1327 (24)	1048 (19)	862 (15)	
>30 kg/m²	873 (23)	600 (16)	930 (24)	725 (19)	675 (18)	

*Includes day cases where admission and discharge are on the same day.

**Table 3:** Distribution of characteristics of 25 014 men and women in 1993–1997 by category of total hospital days 1999–2009

	Categories of total hospital days 1999–2009					
	None (n=6835 27%)	Day case (n=2777 11%)	1–4 nights (n=4950 20%)	5–19 nights (n=5476 22%)	≥20 nights (n=4976 20%)	p Value
Total hospital days, 1999–2009						
Mean±SD	0.0±0.0	1.0±0.0	3.1±1.1	11.4±4.2	65.7±87.7	<0.0001
Median(IQR)	0.0 (0.0–0.0)	1.0 (1.0–1.0)	3.0 (2.0–4.0)	11.0 (8.0–14.0)	44.0 (29.0–73.0)	
Number of admissions, 1999–2009						
Mean±SD	0.0±0.0	1.0±0.0	2.3±1.0	4.1±2.5	11.9±35.0	<0.0001
Median(IQR)	0.0 (0.0–0.0)	1.0 (1.0–1.0)	2.0 (2.0–3.0)	4.0 (2.0–5.0)	7.0 (4.0–12.0)	
Body mass index, kg/m²						
Mean±SD	25.9±3.7	25.9±3.6	26.1±3.8	26.8±4.0	27.1±4.3	<0.0001
Sex (n (%))						
Men	2928 (26)	1230 (11)	2084 (19)	2572 (23)	2414 (21)	<0.0001
Women	3907 (28)	1547 (11)	2866 (21)	2904 (21)	2562 (19)	
Age, years						
Mean±SD	55.4±8.6	56.0±8.5	58.1±8.8	60.6±8.8	64.9±8.2	<0.0001
Age band (n (%))						
<45	481 (47)	179 (17)	197 (19)	133 (13)	42 (4)	<0.0001
45–50	1789 (41)	672 (15)	929 (21)	699 (16)	277 (6)	
50–55	1450 (35)	568 (14)	918 (22)	800 (19)	433 (10)	
55–60	1096 (28)	457 (12)	854 (22)	907 (23)	595 (15)	
60–65	877 (23)	422 (11)	771 (20)	999 (26)	827 (21)	
65–70	659 (17)	289 (8)	741 (19)	987 (26)	1162 (30)	
70–75	400 (13)	160 (5)	456 (15)	799 (26)	1280 (41)	
75–80	83 (12)	30 (4)	84 (12)	152 (21)	360 (51)	
Smoking status (n (%))						
Current	751 (26)	295 (10)	521 (18)	684 (24)	653 (22)	<0.0001
Former	2558 (25)	1118 (11)	1992 (19)	2388 (23)	2367 (23)	
Never	3476 (30)	1351 (12)	2396 (21)	2354 (21)	1892 (16)	
Social class (n (%))						
Professional (1)	599 (35)	210 (12)	320 (19)	343 (20)	252 (15)	<0.0001
Technical (2)	2754 (31)	1045 (12)	1753 (20)	1818 (20)	1579 (18)	
Clerical NM (3.1)	1047 (26)	467 (12)	797 (20)	880 (22)	853 (21)	
Clerical M (3.2)	1397 (25)	603 (11)	1130 (20)	1347 (24)	1149 (20)	
Semiskilled (4)	744 (23)	336 (10)	695 (21)	760 (23)	731 (22)	
Unskilled (5)	163 (19)	76 (9)	167 (19)	204 (24)	250 (29)	
Level of education (n (%))						
Low	1910 (21)	860 (9)	1773 (19)	2237 (25)	2350 (26)	<0.0001
Medium	4122 (31)	1629 (12)	2591 (19)	2734 (21)	2215 (17)	
High	800 (31)	287 (11)	582 (23)	501 (19)	405 (16)	
Body mass index (n (%))						
<24 kg/m²	2225 (32)	885 (13)	1460 (21)	1312 (19)	1103 (16)	<0.0001
24–27 kg/m²	2410 (28)	978 (11)	1761 (20)	1827 (21)	1632 (19)	
27–30 kg/m²	1320 (24)	603 (11)	1058 (19)	1366 (25)	1218 (22)	
>30 kg/m²	873 (23)	310 (8)	658 (17)	958 (25)	1004 (26)	

Table 4 shows the independent relationships using logistic modelling between demographic and behavioural factors in relation to hospital admissions. High numbers of admissions and bed days were positively associated with male sex, age, manual social class, smoking and high BMI while no hospital admissions were inversely associated with these factors. The strongest risk factors for more than 7 admissions were age OR 1.75 (1.67 to 1.82) per 10-year increase, being a current cigarette smoker OR 1.53 (1.37 to 1.71) and BMI ≥30 kg/m² OR 1.41 (1.28 to 1.56). Age was the strongest risk factor for high bed day usage >20 days OR 2.54 (2.44 to 2.65) per 10 years increase in age. Current smoking OR 1.59 (1.44 to 1.77) and BMI ≥30 kg/m² OR 1.54 (1.41 to 1.68) were also important risk factors.

**Table 4:** Multivariable logistic regression of risk factors for no hospital admissions, ≥7 hospital admissions and ≥20 days of hospital stay from 1999 to 2009 in 25 014 men and women aged 40–79 years 1993–1997

	Logistic regression					
	All participants OR (95% CI)	p Value	Men OR (95% CI)	p Value	Women OR (95% CI)	p Value
Outcome of no hospital admissions†						
Female sex	1.11 (1.05 to 1.18)	<0.001	–	–	–	–
Age per 10 years	0.56 (0.54 to 0.57)	<0.001	0.49 (0.47 to 0.52)	<0.001	0.61 (0.58 to 0.64)	<0.001
Non-manual social class	1.29 (1.21 to 1.38)	<0.001	1.35 (1.22 to 1.48)	<0.001	1.24 (1.14 to 1.35)	<0.001
High education level	1.26 (1.18 to 1.35)	<0.001	1.18 (1.06 to 1.32)	0.003	1.32 (1.21 to 1.45)	<0.001
Former or never smoker	1.23 (1.12 to 1.34)	<0.001	1.22 (1.07 to 1.41)	0.004	1.22 (1.08 to 1.39)	0.001
BMI<30 kg/m²	1.25 (1.15 to 1.36)	<0.001	1.22 (1.06 to 1.40)	0.005	1.28 (1.14 to 1.43)	<0.001
Outcome of seven or more hospital admissions†						
Male sex	1.32 (1.22 to 1.42)	<0.001	–	–	–	–
Age per 10 years	1.75 (1.67 to 1.82)	<0.001	1.94 (1.82 to 2.06)	<0.001	1.58 (1.49 to 1.68)	<0.001
Manual social class	1.22 (1.13 to 1.32)	<0.001	1.21 (1.09 to 1.36)	<0.001	1.23 (1.10 to 1.37)	<0.001
Low education level	1.14 (1.05 to 1.24)	0.002	1.05 (0.93 to 1.18)	0.407	1.24 (1.11 to 1.39)	<0.001
Current smoker	1.53 (1.37 to 1.71)	<0.001	1.42 (1.21 to 1.66)	<0.001	1.65 (1.41 to 1.92)	<0.001
BMI >30 kg/m²	1.41 (1.28 to 1.56)	<0.001	1.43 (1.24 to 1.66)	<0.001	1.39 (1.22 to 1.59)	<0.001
Outcome of 20 or more hospital nights†						
Male sex	1.20 (1.12 to 1.28)	<0.001	–	–	–	–
Age per 10 years	2.54 (2.44 to 2.65)	<0.001	2.70 (2.54 to 2.88)	<0.001	2.41 (2.28 to 2.55)	<0.001
Manual social class	1.20 (1.12 to 1.29)	<0.001	1.23 (1.11 to 1.37)	<0.001	1.17 (1.06 to 1.29)	0.003
Low education level	1.17 (1.09 to 1.26)	<0.001	1.07 (0.96 to 1.19)	0.220	1.27 (1.15 to 1.40)	<0.001
Current smoker	1.59 (1.44 to 1.77)	<0.001	1.64 (1.41 to 1.90)	<0.001	1.56 (1.35 to 1.80)	<0.001
BMI >30 kg/m²	1.54 (1.41 to 1.68)	<0.001	1.52 (1.33 to 1.74)	<0.001	1.56 (1.39 to 1.75)	<0.001

†Each variable adjusted for all others listed.

BMI, body mass index.

The demographic and lifestyle factors were used to construct a risk score. Table 5 shows that an increase in the absolute rate of admissions and bed days across score categories was observed in all but the oldest age category. Conversely, the percentage not admitted to hospital over 10 years decreased over increasing risk score categories. In the participants <75 years similar increases in the absolute rates of admissions and bed days were also observed with increasing risk score apart from the highest score categories, though the gradient attenuated with increasing age.

**Table 5:** Absolute percent with no hospital admissions, ≥7 hospital admissions or >20 hospital nights during follow-up 1999–2009 in men and women 40–79 years in 1993–1997

	Outcome rate by score* and age band % outcome rate (outcome frequency/total participants)				
	0	1	2	3	4–5†
Outcome of no hospital admissions (years)					
<55	43 (913/2112)	42 (1445/3425)	34 (862/2537)	33 (365/1107)	28 (62/222)
55–65	32 (425/1314)	28 (749/2681)	22 (487/2176)	19 (223/1170)	16 (40/253)
65–75	23 (229/994)	17 (377/2241)	12 (244/2008)	12 (130/1099)	11 (25/235)
>75	13 (12/95)	11 (28/254)	13 (25/199)	11 (11/96)	0 (0/14)
Outcome of 7 or more hospital admissions (years)					
<55	5 (114/2112)	6 (203/3425)	9 (238/2537)	11 (124/1107)	15 (33/222)
55–65	10 (133/1314)	13 (350/2681)	15 (328/2176)	19 (225/1170)	21 (54/253)
65–75	14 (141/994)	19 (427/2241)	23 (452/2008)	27 (294/1099)	31 (73/235)
>75	21 (20/95)	21 (54/254)	23 (46/199)	22 (21/96)	14 (2/14)
Outcome of 20 or more hospital nights (years)					
<55	5 (105/2112)	7 (230/3425)	10 (244/2537)	11 (127/1107)	14 (32/222)
55–65	13 (173/1314)	16 (439/2681)	18 (397/2176)	25 (289/1170)	30 (76/253)
65–75	26 (262/994)	32 (721/2241)	37 (738/2008)	42 (465/1099)	48 (113/235)
>75	45 (43/95)	50 (127/254)	51 (102/199)	55 (53/96)	43 (6/14)

*Score is defined as the sum of the following binary categories, each contributing one point: male sex, manual social class, low education level, current smoker, body mass index >30 kg/m².

†Scores 4 and 5 combined into a single category due to low numbers having score=5.

Table 6 shows mortality rates over different time periods by age group and risk score. There was a mortality gradient by increasing risk score and the gradient was steeper for the shorter follow-up time. Sensitivity analyses (see online supplementary table) based only on individuals who were at the same postcode throughout the whole duration of this study showed similar results.

**Table 6:** Mortality rates by risk score and age group during before 1993–1998, 1999–2003 and 1999–2009

	Mortality rate* by score† and age band				
	0	1	2	3	4–5‡
Mortality rates before 1993–1998, 1999–2004 and 1999–2009					
<55 years	1993–1999: 0.3	1993–1999: 0.6	1993–1999: 0.5	1993–1999: 0.7	1993–1999: 2.2
	1999–2004: 1.2	1999–2004: 1.4	1999–2004: 2.0	1999–2004: 2.9	1999–2004: 4.4
	1999–2009: 2.2	1999–2009: 3.0	1999–2009: 4.8	1999–2009: 6.4	1999–2009: 7.5
55–65 years	1993–1999: 1.0	1993–1999: 1.3	1993–1999: 1.2	1993–1999: 1.8	1993–1999: 4.2
	1999–2004: 2.9	1999–2004: 4.7	1999–2004: 5.2	1999–2004: 6.8	1999–2004: 12.5
	1999–2009: 7.6	1999–2009: 10.4	1999–2009: 11.5	1999–2009: 15.0	1999–2009: 22.3
65–75 years	1993–1999: 3.8	1993–1999: 5.1	1993–1999: 5.1	1993–1999: 8.0	1993–1999: 7.8
	1999–2004: 8.2	1999–2004: 11.6	1999–2004: 13.4	1999–2004: 17.6	1999–2004: 25.1
	1999–2009: 19.7	1999–2009: 27.7	1999–2009: 30.4	1999–2009: 37.0	1999–2009: 45.1
>75 years	1993–1999: 3.1	1993–1999: 3.8	1993–1999: 5.2	1993–1999: 20.0	1993–1999: 22.2
	1999–2004: 19.4	1999–2004: 24.2	1999–2004: 30.5	1999–2004: 29.2	1999–2004: 33.3
	1999–2009: 48.0	1999–2009: 51.5	1999–2009: 60.0	1999–2009: 46.7	1999–2009: 44.4

*The denominator does not exclude deaths prior to 1999.

†Score is defined as the sum of the following binary categories, each contributing one point: male sex, manual social class, low education level, current smoker, body mass index >30 kg/m².

‡Scores 4 and 5 combined into a single category due to low numbers having score=5.

## Discussion

Our data report hospital usage patterns measured either by the number of hospital admissions or by total bed days, over a 10-year follow-up period in a population of middle aged and older men and women in the UK. We observed that age, male sex, manual social class low education level, current smoking and BMI >30 kg/m² independently predicted multiple admissions and extended time in hospital. A simple five-point risk score constructed using male sex, manual social class, no educational qualifications, current smoking and BMI >30 kg/m², estimated percentages of the cohort in the categories of admission numbers and hospital bed days in stratified age bands with twofold to threefold differences in future hospital use between those with high and low risk scores.

More than half of women under 55 years of age with risk score of zero will expect one or more hospital admission over the next decade but only 5% would have more than 7 admissions or more than 20 nights in hospital. Up to the age of 75 years the number of hospital admissions one might expect increases with the risk score. For those aged 55–65 years only 13% might expect to spend 20 nights in hospital over the next 10 years but this increased to 30% for those with a risk score of four or five. Eighty-seven per cent of men and women over 75 years would expect to be admitted to hospital on one or more occasions over 10 years irrespective of their risk score.

While the trend for increasing hospital use with risk score was not consistent in the oldest age group >75 with the highest risk score, numbers in this group were not large. Possible explanations include substantial differential mortality early on in follow-up resulting in attrition as observed in table 6 so that fewer individuals were at risk of hospital admissions and bed day use over the full 10-year follow-up period.

### Comparison with other studies

Most studies examining hospital usage in the UK are based on hospital data but are limited in their capacity to estimate accurately denominator populations or to assess characteristics prior to hospitalisation and how they may relate to relative or absolute risk of hospital usage prospectively.

The EPIC-Norfolk cohort was recruited from the general population resident in Norfolk and unlike hospital-based studies is able to compare characteristics of hospital attenders and those who did not need to use those services. The period under examination approximately coincides with administrative control by Primary Health Trusts (PCT, 2002–2013) with hospital usage free at the point of delivery under the UK NHS.

Health service usage for study participants resident in the Norfolk area is the responsibility of the East Norfolk PCT irrespective of where in the country the usage occurred. Linkage to the PCT has the advantage of capturing episodes at any UK hospital, not just those in the area. Our study included data from several UK hospitals although the large majority were from Norfolk hospitals. We were able to estimate the probability of hospital admissions and total bed days over a 10-year period according and how they varied according to a range of simple and easily measured demographic and behavioural characteristics generally available in general practice.

A limitation in our study is the lack of information about non-NHS hospital and clinics where study participants paid for treatment. This would include common cosmetic procedures such as the removal of varicose veins and other procedures offered as a private service that may be restricted or not available on the NHS. Data on treatment in private hospitals or clinics were not available to us. It is possible that some of the associations we observed between those in higher social class groups and lower hospital usage are explained by private treatment. However, most serious long-term conditions are treated in NHS hospitals. The differences by sex and BMI we observed were independent of social class and education. It is also possible that individuals may have differentially moved away during follow-up. However, the sensitivity analyses (see online supplementary table) based only on those individuals living in the same post code observed essentially similar results. We have not attempted to examine the reason for admission and simply examined and restricted ourselves to the number of occasions when hospital services were used. The most common reasons for admission were related to diseases of the circulatory system (essential hypertension and chronic ischaemic heart disease being the most common) and diseases of the digestive system (the most common being gastritis, diaphragmatic hernia and diverticular disease). We have also not looked at the survival of those who did or did not use hospital services. Future exploration of these areas will help give us a clearer and more detailed understanding.

While it is not possible to infer causal links between the lifestyle factors and hospital admissions, differences in social class and education may reflect real differences in health status need or demand. Alternatively, thresholds for admission may vary.

In this study, we have identified a range of simple demographic and behavioural indicators that are related to the future probability of cumulative hospital admissions and bed days. The strongest of these are increasing age and male sex. However, the modifiable factors we examined are all strongly associated with hospital usage. Current cigarette smokers were 59% more likely to have 20 of more nights in hospital while those with BMI >30 kg/m² are 54% more likely, indicating an important role of potentially modifiable factors for hospital usage. These and the other simple indicators we have examined are easy to collect and may assist healthcare providers and those planning services to predict future hospital use.
